# Directed Accumulation of Nitrogen Metabolites through Processing Endows Wuyi Rock Tea with Singular Qualities

**DOI:** 10.3390/molecules27103264

**Published:** 2022-05-19

**Authors:** Yang Liu, Zuopin Zhuo, Jing Tian, Bei Liu, Chen Shi, Ruineng Xu, Zilong Guo, Baoshun Liu, Jianghua Ye, Shian He, Wenchun Yang, Maoxing Xu, Lili Sun, Hong Liao

**Affiliations:** 1 Root Biology Center, Fujian Agriculture and Forestry University, Fuzhou 350002, China; liuyang20229300@163.com (Y.L.); zpzhuo66@163.com (Z.Z.); tianjing9808@163.com (J.T.); 18419222809@163.com (B.L.); scyzdq@163.com (C.S.); ruinengxu@fafu.edu.cn (R.X.); cjdxguozilong@163.com (Z.G.); hliao@fafu.edu.cn (H.L.); 2 Wuyi Mountain Tea Industry Research Institute, Wuyishan 354303, China; fjwysmt@163.com (B.L.); jhye1998@126.com (J.Y.); wystea88@163.com (M.X.); 3 Fulian (Wuyishan) Tea Industry Co., Ltd., Wuyishan 354303, China; heshian513@163.com; 4 Wuyishan Shouxi Tea Industry Co., Ltd., Wuyishan 354303, China; 13950672989@163.com

**Keywords:** process, tea quality, N-contained metabolites, metabolome, Wuyi rock tea

## Abstract

The execution of specific processing protocols endows Wuyi rock tea with distinctive qualities produced through signature metabolic processes. In this work, tea leaves were collected before and after each of three processing stages for both targeted and untargeted metabolomic analysis. Metabolic profiles of processing stages through each processing stage of rotation, pan-firing and roasting were studied. Overall, 614 metabolites were significantly altered, predominantly through nitrogen- enriching (N) pathways. Roasting led to the enrichment of 342 N metabolites, including 34 lipids, 17 organic acids, 32 alkaloids and 25 amino acids, as well as secondary derivatives beneficial for tea quality. This distinctive shift towards enrichment of N metabolites strongly supports concluding that this directed accumulation of N metabolites is how each of the three processing stages endows Wuyi rock tea with singular quality.

## 1. Introduction

Tea (*Camellia sinensis*) is one of the most popular beverage crops in the world, not only for its pleasant aroma and taste, but also for its health benefits [1]. Beyond tea variety, growing region and crop management, proper post-harvest processing is regarded as a critical determinant of tea quality [2]. Based on the processing protocols employed, teas are typically divided into the six categories of unfermented green teas, semi-fermented white, yellow and oolong teas, and fully fermented black and dark teas [3]. Different processing technologies endow tea with unique flavors and nutritional attributes, primarily through magnifying the impacts on existing and freshly produced compounds, including both abundant and diffuse metabolites [2,4]. Therefore, the observation of metabolic profiles through tea processing steps may reveal avenues to explore for improving targeted qualities.

A number of reports have identified a portion of quality-related tea metabolites affected by processing [2,4,5,6]. For example, tea aroma may form from precursors of carotenoids, fatty acids, glycosides, amino acids and sugars [2], while taste has been associated with polyphenols, free amino acids, alkaloids, sugars and organic acids [7,8]. In other work with green tea, large amounts of aroma components, especially aldehydes and ketones such as phenylacetaldehyde and β-ionone were produced in gradually increasing quantities throughout processing [4]. Han et al. (2016) [9] introduced varietal comparisons to research on green tea processing methods and found that variety plays a decisive role in the formation of characteristic aromas, while fixation methods have key effects on the strength of green tea taste. For white tea, long-term withering may promote the accumulation of amino acids and thus enhance the freshness of tea soup while also reducing the content of flavor-influencing catechins and flavonoid glycosides [7,10,11]. Fermentation, meanwhile, is critical for altering key components of black tea flavor profiles, with prolonged fermentation noted for dynamically reducing the content of theanine, caffeine and sucrose in analyses of the effects of fermentation duration on targeted metabolites [5,8]. Notably, previous investigations of processing effects on tea quality have mostly focused on green tea and black tea, not other tea categories.

Oolong tea, a semi-fermented tea, is mainly produced in Fujian Province in China [12]. Wuyi rock tea is a kind of oolong tea with a long history of high-quality rankings based on a unique flavor that is highly dependent on a complex empirically driven processing technology that has garnered recognition as a component of China’s National Intangible Cultural Heritage with its three key stages of rotation, pan-firing and roasting [13,14]. In recent years, increasing numbers of studies have investigated processing effects on the quality of Wuyi rock tea [13,14,15,16,17]. Most of these reports focused on changes in tea aroma, with roasting being commonly identified as the key stage to producing the proper roasted aroma [13,14,15,16]. Specifically, 2,5-dimethylpyrazine and 2-ethyl-3,5-dimethylpyrazine have been verified as key aroma-active compounds for peanutty flavor and roasted odor formation during roasting for Wuyi rock tea [15,16]. In addition, leaf rotation has also been regarded as a key processing stage in Wuyi rock tea production during which the concentrations of phenylalanine, tryptophan and 16 other metabolites may significantly increase [17]. Moreover, dramatic changes have also been observed at the pan-firing stage due to the transition of dominant transformation reactions from enzymatic to non-enzymatic browning reactions [6], though scant attention has been paid to this processing stage. Each of the reports on Wuyi rock tea cited above focused on only one processing stage, and thereby provides only fragmentary information.

In this study, fresh leaves of tea cultivar Shuixian were plucked from a tea garden in the Wuyi Mountain region and processed by a tea master. Tea leaf samples were collected after each processing stage, and metabolomic changes were analyzed for each stage through the measurement of both targeted and untargeted metabolites, with the objective of identifying key processing stages and dominant metabolic shifts underlying Wuyi rock tea quality, as well as potential points of improvement, at any stage throughout the multiple steps of processing.

## 2. Results and Discussion

### 2.1. Processing Stages Significantly Changes Metabolite Profiles of Wuyi Rock Tea

**Figure 1:**
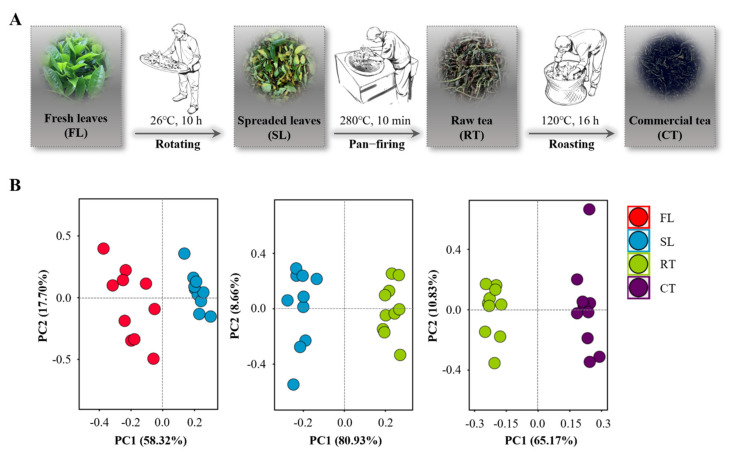
Flowchart of the main processing stages utilized to produce Wuyi rock tea (**A**) and PCA analysis between corresponding tea samples (**B**). The processing stages of Wuyi rock tea include rotation, pan-firing and roasting. The data used for PCA analysis includes the concentrations of conventional quality index compounds and main secondary metabolites measured in the samples of fresh leaves (FL), spread leaves (SL), raw tea (RT) and commercial tea (CT).

In general, the process stage is regarded as the main factor affecting the quality of Wuyi rock tea [8]. The technology of processing Wuyi rock tea is quite intricate and closely dependent on empirical observations throughout the key stages of rotation, pan-firing and roasting of tea leaves (Figure 1A) [8,16]. The PCA results from five conventional quality indexes and seven targeted secondary metabolites revealed that the tea samples were separated significantly during each of the rotation, pan-firing and roasting stages and that the first two principal components accounted for 76.02%, 89.59% and 76.00% of the respective total variation (Figure 1B). These PCA results verify the importance of each processing stage for the quality production of Wuyi rock tea.

**Figure 2:**
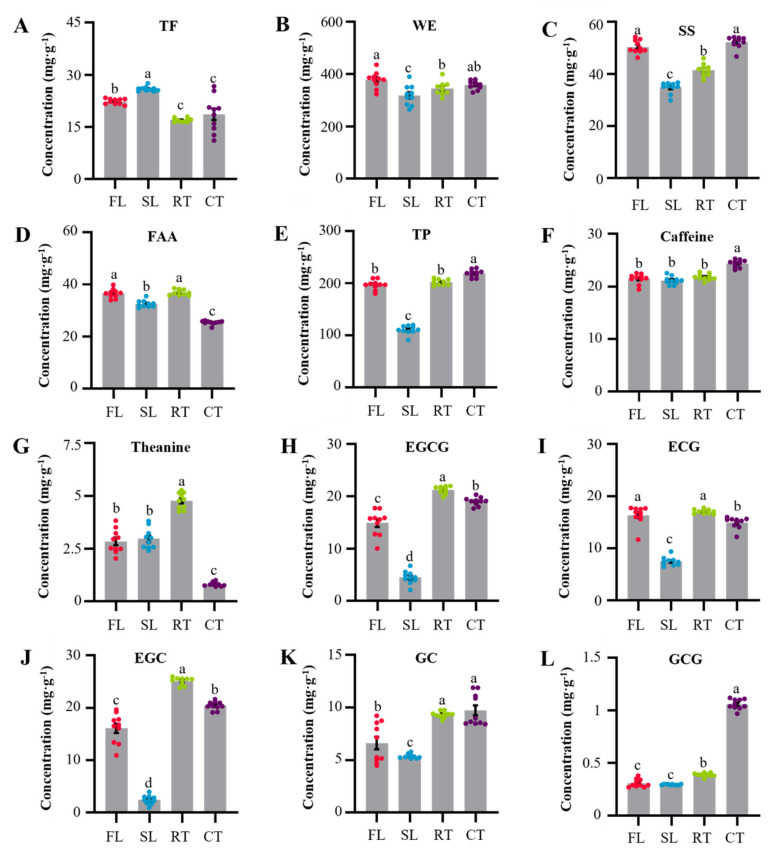
Dynamic changes in the concentrations of conventional quality index compound categories (**A**–**E**) and main secondary metabolites (**F**–**L**) through the processing stages of Wuyi rock tea. Conventional quality index compound categories include total flavonoids (TF), water extracts (WE), soluble sugars (SS), free amino acids (FAA) and tea polyphenols (TP). The main secondary metabolites include caffeine, theanine, epigallocatechin gallate (EGCG), epicatechin gallate (ECG), epigallocatechin (EGC), gallocatechin (GC) and gallocatechin gallate (GCG). FL: fresh leaves; SL: spread leaves; RT: raw tea; CT: commercial tea. The values are shown as means ± standard errors (SEs), (*n* = 10). Different letter(s) indicate significant differences among process stages at the *p* = 0.05 threshold level.

Conventional indexes used for evaluating tea quality primarily focus on TF, WE, SS, FAA and TP [12]. Throughout processing, TF increased significantly by 15.98% up to a maximum after the rotation stage, from which it declined dramatically by 34.55% down to the lowest observed concentrations in the subsequent pan-firing stage (Figure 2A). In contrast, WE, SS, FAA and TP exhibited respective declines of 15.69%, 30.97%, 10.90% and 43.76% during rotation, and respective increases of 8.35%, 19.29%, 13.38% and 82.40% with pan-firing (Figure 2B–E). In the rotation stage, oxidative reactions appeared to be proceeding vigorously with intermittent rotational motion through the activation of oxidases such as polyphenol oxidase and peroxidase [7,8]. After partial fermentation, some tea polyphenols were polymerized into theaflavins and thearubigins [2], leading to a sharp decrease of TP and an increase of TF (Figure 2A,E). Flavonol glycosides and flavonoid glycosides were also promoted through the acceleration of oxidative reactions of flavonols and flavonoids with glycosyl groups [18] which further resulted in the increase of TF and the reduction of SS (Figure 2A,C). Subsequently, hydrothermal reactions played a dominant role in the pan-firing stage under the conditions of high temperature and moisture [12,19]. During this stage, the concentrations of TF were reduced through flavonoid glycoside hydrolysis (Figure 2A) [12], while those of SS and FAA increased via the hydrolysis of cellulose and protein, respectively (Figure 2C,D) [20].

The most striking changes in these experiments were observed for TP, which declined by over 40% with rotation and increased more than 80% during pan-firing. These observations clearly indicated that phenolic metabolism was active during these two stages (Figure 2E). Since about 60%~80% of TP in tea samples are typically catechins, which are primarily found to be EGCG, ECG, EGC, GC and GCG [11], the concentrations of these compounds were further assessed. Consistent with the changes in TP, the concentrations of EGCG, ECG, EGC and GC sharply declined with rotation and significantly increased in pan-firing (Figure 2H–K). On the other hand, the concentrations of GCG did not change significantly with rotation (Figure 2L). The trend in TP changes corresponded closely with changes in EGCG, ECG, EGC and GC (Figure 2H–K), indicating these compounds function as the dominant catechins contributing TP levels in processed tea. It has also been reported that the bioavailability of EGCG, ECG and EGC may be higher than that of GC and GCG, which are respective epimerization products of EGC and EGCG in high-temperature roasting [12,19].

As the main contributor to FAA concentrations, theanine exhibited changes that were a little different from FAA in the rotation stage. Specifically, the concentrations of FAA slightly dropped, and theanine did not change significantly in the rotation stage. However, the concentration of theanine and FAA significantly increased in pan-firing before dramatically dropping again in roasting (Figure 2D,G). The increases in FAA and theanine in pan-firing may be due to the hydrolysis of protein and theanine glycosides, respectively [17,20]. Prolonged heat in roasting promotes Maillard reactions between amino acids and reduced sugars, which reduces the concentrations of FAA and theanine [15].

Caffeine, as one of the most important alkaloids in tea, remained stable during rotation and pan-firing (Figure 2F). Then, with prolonged exposure to high temperatures in roasting, caffeine appeared to be released from complexes to produce an 11.86% increase in the concentration of caffeine in CT compared with RT (Figure 2F) [13,20].

Taken together, the results above suggest that processing significantly changes metabolite profiles and overall quality of Wuyi rock tea. In addition, both conventional tea quality indexes and targeted secondary metabolites exhibited consistent dynamic changes through the process stages, suggesting that consistent and predictable metabolomic shifts occur during the process of manufacturing Wuyi rock tea.

### 2.2. N-Associated Pathways Exhibited Obvious Enrichment through Wuyi Rock Tea Processing

**Figure 3:**
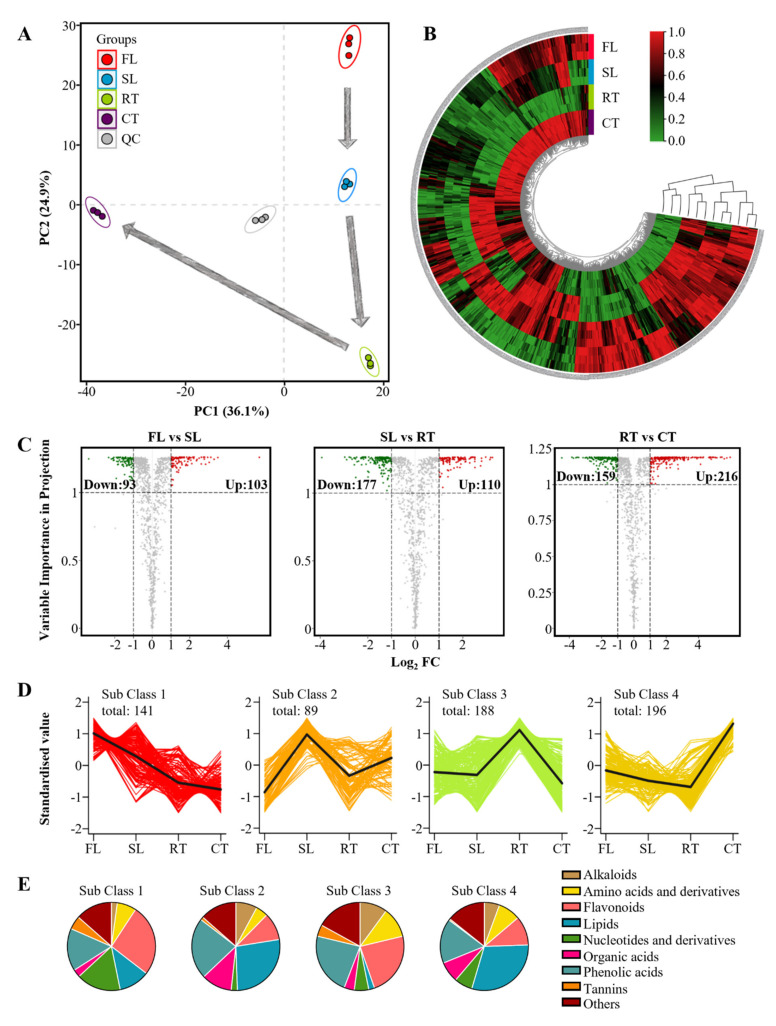
Analysis of metabolites significantly altered through processing of Wuyi rock tea as identified in untargeted metabolomics: (**A**) PCA plots; (**B**) heat map and cluster analysis; (**C**) volcano plot maps; (**D**) trends in the abundance of significant metabolite clusters across processing stages, with metabolite clusters delimited in K-means cluster analysis of metabolites displaying significant variation in concentration across processing stages; (**E**) Diagrams of significant metabolite subclasses grouped according to K-means clustering of metabolites altered across processing stages. FL: fresh leaves; SL: spread leaves; RT: raw tea; CT: commercial tea.

Metabolomics may be an effective means to comprehensively investigate the metabolites changed during tea processing stages [5,8,11]. To further investigate the consistency processing outcomes for Wuyi rock tea, untargeted metabolomics were conducted through ESI-Q TRAP-MS/MS. These procedures yielded a total of 1143 metabolites, which were used to perform PCA analysis of the different process stages (Figure 3A). In this analysis, QC samples clustered near the origin, implying that sample measurement is highly repeatable and stable (Figure 3A). In addition, the separation of tea samples from different stages was readily apparent in PCA plots with variances of 36.1% and 24.9% observed for PC1 and PC2, respectively (Figure 3A). Additionally, CT samples separated from the FL, SL and RT samples in the direction of PC1, implying a dramatic impact of roasting (Figure 3A). Previously, it has been reported that roasting significantly changes flavor components in rock tea with impacts including production of substances associated with roasting flavor, such as 2,5-dimethylpyrazine, 2-ethyl-3,5-dimethylpyrazine and tea pyrrole [13,15,16]. In the current work, 614 metabolites exhibiting changes in concentration across processing stages were selected and clustered in a heat map (Figure 3B). Consistent with the PCA results, CT samples were definitely disparate clusters of FL, SL and RT profiles (Figure 3B). This result further demonstrates that roasting is the most influential stage in altering the quality of Wuyi rock tea as commonly reported elsewhere [13,14,15,16].

In volcano maps, significantly altered metabolites decreased by 61.7% with pan-firing and increased by 52.6% and 57.6% in the rotation and roasting stages, respectively (Figure 3C). In addition, the largest number of differential metabolites were generated after the roasting stage (Figure 3C), which also implied that roasting was a critical stage in the processing of Wuyi rock tea. To further investigate the metabolic pathways affected at each of the processing stages, K-means cluster analysis was conducted. In this analysis, 614 altered metabolites were mainly divided into four subclasses containing the 141, 89, 188 and 196 metabolites that were observed in their highest concentrations in FL, SL, RT and CT (Figure 3D) samples, respectively. Each of these subclasses exhibited unique patterns in diagrams of their compound families (Figure 3E).

Among the tested classes of secondary compounds, phenolic acids were the most stable type, with these compounds accounting for 15.6–22.87% of the measured metabolites within the four subclasses (Figure 3E). As a class of aromatic compounds containing carboxyl and hydroxyl groups, phenolic acids may produce derivatives or isomers with important flavor roles in tea processing [11]. Flavonoids and tannins were much more prevalent in Subclasses 1 and 3 than in Subclasses 2 and 4, with tannins becoming extremely rare in Subclass 4, suggesting that flavonoids and tannins are dramatically reduced upon completion of processing (Figure 3D,E).

The types and ratios of flavonoids and tannins determine the bitterness and astringency of tea [21]. Thus, astringency may be reduced when flavonoid glycosides decrease with increasing temperatures [11,20]. Alkaloids, major contributors to bitterness, appeared in Subclasses 2, 3 and 4, which indicates that these secondary metabolites formed gradually during processing (Figure 3D,E) [11]. Among alkaloids, caffeine, which is typically the alkaloid present in the highest concentrations, may form fresh flavor complexes with theaflavins through hydrogen bonding [12]. This suggests that alkaloids generated during processing stages might positively impact flavor profiles.

Lipids were most prevalent in Subclass 2 (26.97%) and Subclass 4 (30.10%) in our study (Figure 3E), suggesting that rotation and roasting promote lipid production. Consistently, others report that more than half of initial lipid fractions in oolong tea degraded in rotation and roasting, primarily from storage and membrane lipid sources with fatty acids concomitantly increasing in concentration [12,13]. During the rotation stage, activation of lipid degrading enzymes and lipoxygenases might explain the accelerated degradation of lipids into free fatty acids, while in the roasting stage, the thermal degradation of lipids is explainable as a product of the high temperature conditions [1,6,14].

**Figure 4:**
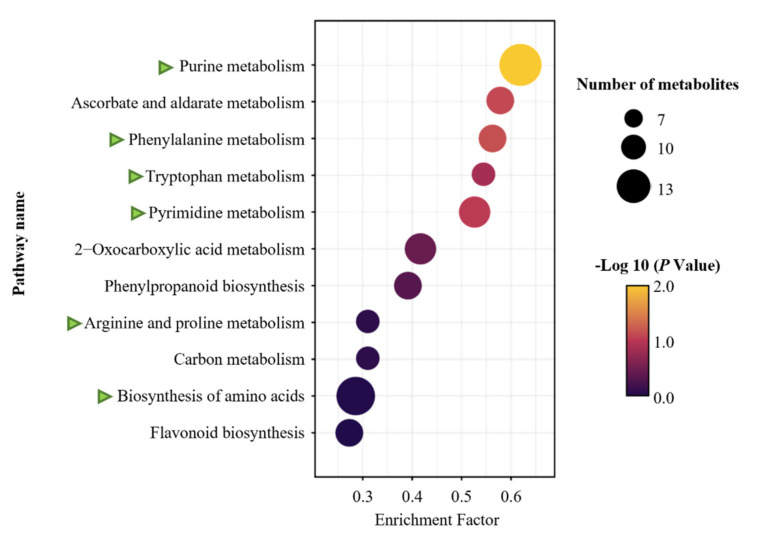
KEGG enrichment analysis of untargeted metabolites that were significantly altered between FL and CT. Green triangles indicate pathways associated with N metabolism.

To further determine the key pathways associated with altered metabolites, KEGG enrichment analysis was conducted between the metabolites identified in FL and CT samples. The results showed that N pathways were the most highly enriched among those associated with altered metabolites, as indicated by green arrows in Figure 4. The affected N pathways include those functioning in purine, phenylalanine, tryptophan, pyrimidine, arginine and proline metabolism, as well as others acting in the biosynthesis of amino acids. Nucleotides generated from nucleotide metabolism are known to be important precursors of caffeine, theobromine and theophylline, all of which may effectively influence tea flavor [12]. For example, adenine has been reported as a precursor for caffeine synthesis, and hypoxanthine nucleotides can increase the umami taste of tea infusions [12,22]. In our results, both purine and pyrimidine metabolism associated with alkaloid generation were significantly enriched (Figure 4), demonstrating that alkaloids are important components of rock tea processing. Beyond nucleotides, amino acids have also been documented as carriers of tea umami flavoring [22]. Plus, Wang et al. (2018) have found that amino acid metabolism is critical in tea processing [10], with phenylalanine identified as an important precursor for the production of flavonoids and volatile benzene derivatives. Tryptophan is another important precursor known for roles in indole alkaloid production, which yields familiar aroma components in oolong tea and contributes to fruit and floral flavors [4,12]. Indoles can be released in the processing stages of oolong tea, both through the oxidation of tryptophan indolease at picking, as well as through thermal degradation of the Amadori compound L-tryptophan in the Maillard reaction at the roasting stage [1,13,23].

Collectively, N–related metabolites appear to have undergone significant changes during the processing stages studied herein, which might be critical for the production of the unique flavors attributed to Wuyi rock tea.

### 2.3. N Metabolites Increase Dramatically in the Roasting Stage

**Figure 5:**
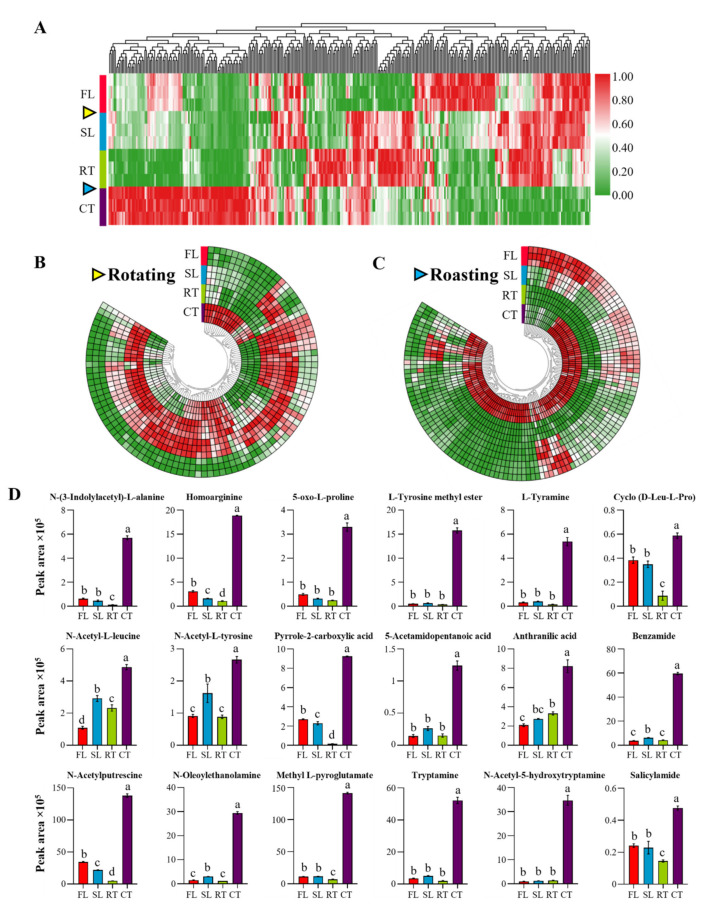
Dynamic changes in the abundance of N-containing metabolites across the processing stages of Wuyi rock tea production: (**A**) heat map of all altered N metabolites; (**B**,**C**) dynamic profiles of N metabolites that were significantly increased in the rotation and roasting stages, respectively; (**D**) changes of key metabolites that significantly increased in the roasting stage from (**C**). The values are shown as means ± standard errors (SEs), (*n* = 3). Yellow and blue triangles indicate the rotation and roasting stages, respectively. Different letters indicate significant differences among different processing stages at the *p* = 0.05 threshold level. FL: fresh leaves; SL: spread leaves; RT: raw tea; CT: commercial tea.

In previous reports, N metabolites have been identified as critical components of tea quality, with impacts noted on aroma, flavor and nutritional aspects of processed tea [24,25]. To comprehensively elucidate changes in N metabolite profiles accumulating through the three processing stages, 342 N metabolites were selected for analysis from the full list of 614 altered metabolites (Figure 5A), Observations of these N metabolites revealed that they increased significantly in abundance during the rotation and roasting stages (Figure 5B,C). Specifically, 34 lipids, 17 organic acids, 32 alkaloids and 25 amino acids and amino acid derivatives were rapidly enriched during roasting (Figure 5C and Figure S1A; Supplementary Materials Table S1).

Lipids are important hydrophobic compounds in tea that have been found to be changed through diverse oxidation and thermal degradation pathways during processing [6,26]. Notably, the N-containing lipids that increased an average of nearly five fold during the roasting stage were all lysophosphatidylcholine (LPC) derivatives (Figure S1A; Table S1). Li et al. (2021) [6] reported that drying and pan-firing were the most critical processing stages for affecting lipid metabolomics, and eight LPCs did exhibit significant concentration changes during green tea processing, which indicates that high temperature might be a key factor affecting the production of LPCs. This could explain why a mass of LPCs changed in the roasting stage in our study (Figure S1A; Table S1). Three LPCs have also come to be regarded as key metabolites associated with the hypoglycemic functions of dark tea [27]. Along with the formation of LPCs, lipids have also been associated with the rupture of fatty acid chains [28]. In this study, 61.8% of the 34 LPCs significantly enhanced in roasting contained unsaturated fatty acids (Table S1). Furthermore, unsaturated fatty acids such as linolenic acid, are important precursors in the formation of aromatic compounds in tea [1]. In addition, floral, fruity and woody components of Wuyi rock tea aromas have been associated with oxidized products of unsaturated fatty acids such as hexanal, valeraldehyde, nonanal, methyl jasmonate and nerolid [1,13]. Therefore, the 34 lipids enriched in roasting identified herein might contribute to both the unique taste and the aroma of Wuyi rock tea.

Organic acids are another important flavor component that are usually released in the heating steps of tea processing [6]. In this study, pyrrole-2-carboxylic acid and 5-acetamidopentanoic acid were the two key organic acids observed to increase with roasting by 61 and 7.2 fold, respectively (Figure 5D and Figure S1A; Table S1). Pyrrole compounds produced during roasting have been reported as largely responsible for the baked aromas of rock tea [16]. As a precursor of pyrrole, the pyrrole-2-carboxylic acid identified in tea samples observed here might play an important role in the formation of a baked aroma for Wuyi rock tea. Additionally, pyrrole-2-carboxylic acid derivatives are known to function as an anti-browning agent in pineapple [29]. Among the organic acids accumulating during the roasting stage in this study (Figure S1A; Table S1), methyl anthranilate is known as a main contributor to fruit flavors, while anthranilic acid, 2-formylamino-benzoic acid and 2-amino-3-methoxybenzoic acid are important precursors for benzyl benzoate, a key metabolite noted for contributing a jasmine fragrance [30]. Taken together, therefore, the profile of organic acids enriched in roasting might form baked aromas, fruit flavors, a jasmine fragrance and brown coloration in Wuyi rock tea.

Alkaloids are a prominent family of N metabolites that includes among its members purine alkaloids, organic amines, indole alkaloids, and so on [31]. Purine alkaloids such as caffeine, theobromine and theophylline are important constituents of tea that are mainly derived from the metabolism of purine nucleotides [12]. In our study, the concentrations of 32 alkaloids increased in roasting (Figure 5D and Figure S1A; Table S1), which led to significant enrichment of purine metabolism among identified KEGG annotated metabolic pathways (Figure 4). Consistently, a large number of nucleotide derivatives were copiously produced (Table S1), possibly due to accelerated degradation of nucleotides under extremely high temperature conditions.

As a frequently used medicine and health promoting ingredient [32], salicylamide was remarkable, increasing more than two fold with roasting (Figure 5D). It is also worth noting that oxygen deprivation may lead to tryptamine accumulation during roasting [33]. In the present work, roasting led to over 18-fold increases in tryptamine and N-acetyl-5-hydroxytryptamine (Figure S1A), two indoleamines derived from tryptophan that can serve as precursors of serotonin and melatonin, respectively [34]. At the same time, serotonin and melatonin are widely considered to be important signaling molecules with wide-ranging physiological effects in both plants and animals, where they may act as anti-depressants, sleep aids and promoters of cancer cells apoptosis [34,35]. Relatively high concentrations of serotonin have been found in many fruits, such as bananas, kiwis, pineapples, tomatoes, grapes and coffee beans, while the present study demonstrates the presence of serotonin in tea. Overall, the enrichment of alkaloids in roasting might increase the health-promoting benefits of Wuyi rock tea, while also opening avenues for ideas to further develop and utilize indoleamine in Wuyi rock tea processing.

Amino acids and amino acid derivatives underwent significant changes during the roasting stage, with the result that profiles were dominated by the accumulation of glutamic acid, alanine and serine (Figure 5D and Figure S1A; Table S1). Derivatives of alanine and tyrosine and potential products of Maillard reactions, N-(3-indolylacetyl)-L-alanine, L-tyrosine methyl ester and L-tyramine also increased with roasting 47.19, 34.07 and 40.06 fold, respectively (Figure 5D). In addition, large amounts of proline, arginine, leucine and isoleucine derivatives were produced in roasting (Table S1). The derivatives of proline and arginine can contribute to the aroma of toasted bread and toasted sucrose, respectively, and those of leucine and isoleucine may produce the aroma of grilled cheese [36].

In short, a large amount of N metabolites enriched dramatically in the roasting stage of tea processing, including representatives of lipids, organic acids, alkaloids and amino acids, all of which might contribute to the baked, sweet, pleasant aromas, tastes and health promoting aspects of Wuyi rock tea.

### 2.4. Precursors of N Metabolites Enriched in Roasting Might Be Produced in Rotation and Pan-Firing Stages

Most of the N-containing compounds enriched in roasting were likely formed in Maillard reactions with amino acids and reducing sugars as precursors [9,12,14,26]. Interestingly, amino acids accumulating in the rotation stage, and monosaccharides increasing in the pan-firing stage might be the precursors for N metabolites enriched in roasting (Figure 5 and Figure S1).

Nearly one-third of the metabolites that accumulated significantly during the rotation were amino acids and amino acid derivatives, including 15 basic amino acids (Figure 5B and Figure S1B). This result is consistent with previous reports that amino acid concentrations increase significantly during rotation [8,17]. A possible explanation is that collisions between leaves stimulates the activation of oxidases and hydrolases, which simultaneously promote reactions of amino acids, flavanols, flavonol glycosides and theaflavins in tea leaves, where they might contribute to specific aromas and flavors [1,8].

The concentrations of phenylalanine, tryptophan and tyrosine in SL increased 3.7, 2.1 and 1.7 fold, respectively, in comparison with their concentrations in FL (Figure S1B). It remains possible that these amino acid increases are promoted by protease activity during the rotation processing stage [7,17].

Phenylalanine, tryptophan and tyrosine are often considered as the amino acids that characterize the fermentation stage of Wuyi rock tea production [17]. Phenylalanine can be degraded to produce phenylacetaldehyde, a volatile compound with a sweet smell of honey [4]. A precursor of indole with known positive effects on the aroma of oolong tea originated from members of tryptophan metabolic pathways [12,23]. Additionally, tyrosine has been reported as a precursor of dopamine, which functions as a neurotransmitter and regulator of Parkinson’s disease and schizophrenia [37]. Therefore, increasing production of amino acids during the rotation stage might provide substrates for the production of aroma and human health-promoting substances.

Most of the monosaccharides identified in the tea leaves observed herein decreased sharply in abundance during roasting. Exceptions include D-fructose-1,6-biphosphate, D-galactose and D-sedoheptuiose 7-phosphate, all of which might contribute to sweet tastes in commercial tea (Figure S1C). The concentrations of 14 monosaccharides decreased by more than 50%, possibly due to participation in Maillard reactions with amino acids (Figure S1C). Across the three stages of rock tea processing, concentrations of D-fructose, D-glucose and D-mannose all decreased fairly uniformly (Figure S1C). Glucose is a preferred substrate in Maillard reactions, where it can react with alanine and tyrosine to generate caramel flavors under high temperature roasting conditions. Additionally, glucose may also directly react with theanine to generate volatile pyrazine, pyrrole and furan derivatives at 150 °C [1,15], which might explain the sharp decrease of theanine observed in roasting (Figure 2G). Interestingly, many monosaccharides increased dramatically in short-term high temperature fixation procedures, including rhamnose and L-fucose, which increased by 1.75 times and 2.09 times, respectively. This short-term increase in monosaccharides might be due to the hydrolysis of polysaccharides at high temperatures.

## 3. Materials and Methods

### 3.1. Collection of Tea Samples

Tea cultivar Shuixian was employed in this study. The experimental tea garden was located in Yanzike of Wuyi Mountain (Fujian, China). In May 2020, fresh tea leaves were plucked according to local standards, with three old leaves and one new leaf processed by one master who was awarded as the Inheritors of intangible cultural heritage in China, with extensive experience in tea making craftsmanship. All fresh tea leaves were plucked and transported to the tea makers’ factory for tea processing. The fresh tea leaves (FL) were sampled immediately when arriving at the destinations. The FL were then withered for 3.5 h and rotated for about 10 h at 26 °C prior to laying out spread leaves (SL) and pan-firing at 280 °C for 10 min. Raw tea (RT) was obtained after quick drying to a moisture content below 6%. Finally, commercial tea (CT) was produced via roasting of RT on a charcoal fire at 120 °C for 16 h. A total of 10 replicate samples were collected, processed and analyzed at each of the FL, SL, RT and CT stages for a total of 40 tea leaf samples that were each hot-fixed and ground for determination of conventional quality indexes and core secondary metabolites. Additionally, 3 replicates of samples were also freeze-dried and analyzed for untargeted metabolites.

### 3.2. Determination of Conventional Quality Indexes

Water extracts (WE), polyphenols (TP), soluble sugars (SS) free amino acids (FAA) and total flavonoids (TF) are five conventional indexes of tea quality [12]. According to the Chinese National Standard (GB/T 8305-2013), WE were collected by boiling 2 g tea powder in 300 mL distilled water for 45 min in a 500 mL erlenmeyer flask prior to shaking for 10 min and immediately filtering suspended particles. Filtered solutions were ready for TP, SS and FAA analysis, while residues were ovendried and weighed to calculate WE concentrations.

The TP, SS and FAA values were determined according to previously described methods [38]. Briefly, to determine TP, 1 mL of the ME extracted above was mixed with 5 mL 10% (*v*/*v*) Folin-Ciocalteu phenol solution and 4 mL 7.5% (*v*/*v*) sodium carbonate solution in a 10 mL colorimetric tube, mixed evenly and equilibrated at room temperature for 1 h before measuring TP in a spectrophotometer (UV-1780, Shimadza, Kyoto, Japan) at the absorbance of 765 nm. To detect SS, 1 mL ME was mixed with 4 mL anthrone reagent (Solarbio Science Technology, Beijing, China), heated in boiling water for 15 min, put in an ice bath to cool down quickly and observed in a spectrophotometer (UV-1780, Shimadza, Kyoto, Japan) for absorbance at 620 nm. To detect FAA, 1 mL ME was mixed with a 0.5 mL phosphate buffer and 0.5 mL ninhydrin reagent (Solarbio Science Technology, Beijing, China), heated in boiling water for 15 min, put in an ice bath to cool and then observed in a spectrophotometer (UV-1780, Shimadza, Kyoto, Japan) for absorbance at 570 nm.

The determination of total flavonoids (TF) followed previously described methods [39]. Here, 0.5 mL ME, 1.5 mL 95% (*v*/*v*) methanol aqueous solution, 0.1 mL 10% (*m*/*v*) AlCl_3_, 0.1 mL 1 M sodium acetate anhydrous and 2.8 mL distilled water were mixed evenly in a 10 mL colorimetric tube, incubated at room temperature in the dark for 1 h, and measured in a spectrophotometer (UV-1780, Shimadza, Kyoto, Japan) for absorbance at 415 nm.

### 3.3. Determination of Targeted Secondary Metabolites

In total, seven secondary metabolites were quantified, including caffeine, theanine and five catechins, epigallocatechin gallate (EGCG), epicatechin gallate (ECG), epigallocatechin (EGC), gallocatechin (GC) and gallocatechin gallate (GCG), according to methods modified from Han et al., 2016. Briefly, 50 mg of sample powder was mixed with 1 mL 70% (*v*/*v*) methanol solution in a 1.5 mL centrifuge tube. After vortexing, the mixed solution was sonicated at 25 °C for 30 min, and then centrifuged at 12,000 g for 10 min. The supernatant was then filtered through a 0.22 μm filter (Millipore, Darmstadt, Germany) before detecting candidate metabolites in high performance liquid chromatography (HPLC-1260, Agilent, Santa Clara, CA, USA) using the ZORBAX SB-Aq column (4.6 × 250 mm, 5 μm, Agilent, Santa Clara, CA, USA). The mobile phase consisted of solvent A and B, which were 0.05% phosphoric acid solution (Sigma-Aldrich, Shanghai, China) prepared with Milli-Q water (Millipore, Billerica, MA, USA) and 100% acetonitrile solution (Merck, Darmstadt, Germany), respectively. Metabolites were separated with a gradient-changed mobile phase set to 88% A and 12% B in the initial 18 min, 84% A and 16% B in the next 2 min and finally 84% A and 16% B during the last 15 min. The flow velocity was 1 mL/min. The column temperature was set to 30 °C. The injection volume was 20 μL. The detection wavelength was 196 nm for theanine, and 278 nm for caffeine and catechins. The standards used for quantification of caffeine, theanine and the five measured catechins were purchased from Yuanye Bio-Technology Co., Ltd. (Shanghai, China).

### 3.4. Determination of Untargeted Metabolites

Untargeted metabolomic analysis of samples followed previously described methods [40]. Here, 100 mg sample powder was dissolved in 1.2 mL 70% (*v*/*v*) methanol, vortexed and kept at 4 °C for 12 h. After centrifugation at 12,000 g for 10 min, the supernatant was filtered through a 0.22 μm filter and analyzed via an ultra-high performance liquid chromatography electrospray ionization tandem mass spectrometry (UPLC-ESI-MS/MS) system (UPLC, Nexera X2, Shimadzu, Kyoto, Japan; MS/MS, Applied Biosystems 4500 Q TRAP, Thermo Fisher Scientific, Waltham, MA, USA). The UPLC column was an SB-C18 model (2.1 × 100 mm, 1.8 μm, Agilent, Santa Clara, CA, USA). The mobile phase consisted of solvent A Milli-Q water and solvent B acetonitrile, both of which contained 0.1% formic acid. In the running program, the proportion of solvent B was 5% at the beginning, increasing linearly to 95% within 9 min, holding at 95% for 1 min, decreasing linearly to 5% within 1.1 min and kept at 5% for 2.9 min until terminating flow. The flow velocity was 0.35 mL/min with the column oven set to 40 °C. The injection volume was 4 μL.

Linear ion trap (LIT) and triple quadrupole (QQQ) mass spectrometry were conducted with the positive and negative ion modes operating in an AB4500 Q TRAP UPLC/MS/MS system equipped with an ESI Turbo Ion-Spray interface. Analyst 1.6.3 software (AB Sciex, Boston, MA, USA) was used for processing. The ESI ion source was running with the turbo spray set at 550 °C. The spray voltages of positive and negative ion modes were 5500 V and −4500 V, respectively. The ion sources of gas I (GSI), gas II (GSII) and curtain gas (CUR) were 50, 60 and 25.0 psi, respectively. A mixture of 10 and 100 μM polypropylene glycol solutions were used for instrument tuning and mass calibration in QQQ and LIT modes, respectively.

### 3.5. Data Analysis

Data are exhibited as means ± standard errors (SEs). The significance of statistical differences among different processing steps was tested by one-way analysis of variance (ANOVA) and Duncan’s multiple range test using SPSS software (Version 19.0.0, International Business Machines Corporation, Chicago, America). The prcomp function in R (Version 3.6.2, Comprehensive R Archive Network, Shanghai, China) was used for principal component analysis (PCA). Significantly impacted metabolites were identified as those with variable importance in projection (VIP) and absolute value of log_2_ fold change (FC) values of ≥1. VIP values were obtained by orthogonal partial least square discriminant analysis (OPLS-DA) using the R package MetaboAnalystR. Significantly impacted metabolites were annotated through homology to KEGG compound database entrants, mapped to KEGG pathways, and fed into metabolite set enrichment analysis. Heat maps were constructed using log transformed (log_2_) and normalized data in the TBtools software (Version 1.068, https://github.com/CJ-Chen/TBtools (accessed on 15 April 2022), Guangzhou, China) [41].

## 4. Conclusions

**Figure 6:**
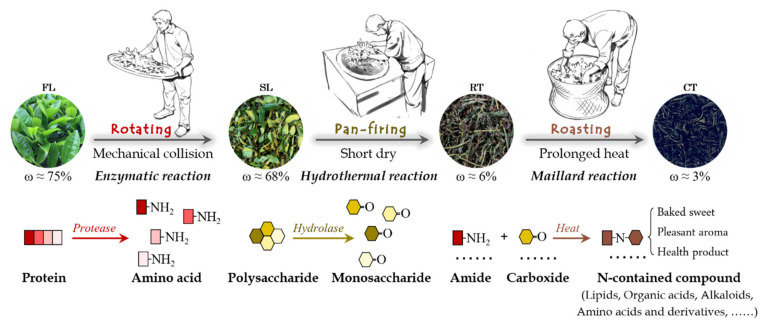
Model of Wuyi rock tea processing stages and mechanisms proposed to underlie the accumulation of N metabolites. In the rotation stage, proteins can be degraded to various amino acids and their derivatives through mechanical collisions spurred in leaf rotation. Then, pan-firing promotes hydrolysis of polysaccharides in high temperature and moisture conditions. Subsequent Maillard reactions occurring in the roasting stage promote a large increases in N-contained metabolites via the combination of amidogen and carbonyl compounds. The resulting N metabolites increase the quality of Wuyi rock tea by imparting specific aromas, flavors and health-promoting characteristics. ω: moisture percentage of samples.

In summary, processing stage has a decisive contribution to Wuyi rock tea quality, with metabolites tending to be enhanced in steps through the rotation, pan-firing and roasting stages. The orientated accumulation of N-contained compounds appears to be vital for developing the unique qualities of Wuyi rock tea (Figure 6), with production occurring primarily in the roasting stage, apparently through Maillard reactions. Amino acid substrates for Maillard reactions are generated in the rotation stage, while monosaccharides are released during the pan-firing stage. Therefore, the three key processing stages for Wuyi rock tea production, namely rotation, pan-firing and roasting, coordinately contribute the aromas, flavors and health-promoting aspects to the final Wuyi rock tea product.

## Data Availability

Not applicable.

## Supplementary Materials

The following supporting information can be downloaded at: https://www.mdpi.com/article/10.3390/molecules27103264/s1. Supplementary Figure S1: Heat maps of specific metabolites. (A) N metabolites exhibiting significant increases in concentration during the roasting stage. Information on specific N metabolites graphed above is detailed in Supplemental Table S1. (B) Amino acids significantly increased in the rotation stage. (C) Monosaccharides significantly altered through the three processing stages of Wuyi rock tea production. FL: fresh leaves; SL: spread leaves; RT: raw tea; CT: commercial tea. Table S1: Detailed information on metabolites exhibited in Figure S1A.

## Sample Availability

Samples of the compounds are not available from the authors.

## References

[1] Ho C.T., Zheng X., Li S.M. (2015). Tea aroma formation. Food Sci. Hum. Well..

[2] Feng Z.H., Li Y.F., Li M., Wang Y.J., Zhang L., Wan X.C., Yang X. (2019). Tea aroma formation from six model manufacturing processes. Food Chem..

[3] Zheng W.J., Wan X.C., Bao G.H. (2015). Brick dark tea: A review of the manufacture, chemical constituents and bioconversion of the major chemical components during fermentation. Phytochem. Rev..

[4] Wang C., Lv S.D., Wu Y.S., Lian M., Gao X.M., Meng Q.X. (2016). Study of aroma formation and transformation during the manufacturing process of Biluochun green tea in Yunnan Province by HS-SPME and GC-MS. J. Sci. Food Agric..

[5] Tan J.F., Dai W.D., Lu M.L., Lv H.P., Guo L., Zhang Y., Zhu Y., Peng Q.H., Li Z. (2016). Study of the dynamic changes in the non-volatile chemical constituents of black tea during fermentation processing by a non-targeted metabolomics approach. Food Res. Int..

[6] Li J., Hua J.J., Yuan H.B., Deng Y.L., Zhou Q.H., Yang Y.Q., Dong C.W., Zeng J., Jiang Y.W. (2021). Investigation on green tea lipids and their metabolic variations during manufacturing by nontargeted lipidomics. Food Chem..

[7] Dai W.D., Xie D.C., Lu M.L., Li P.L., Lv H.P., Yang C., Peng Q.H., Zhu Y., Guo L., Zhang Y. (2017). Characterization of white tea metabolome: Comparison against green and black tea by a nontargeted metabolomics approach. Food Res. Int..

[8] Chen S., Liu H.H., Zhao X.M., Li X., Shan W.N., Wang X.X., Wang S.S., Yu W.Q., Yang Z.B., Yu X.M. (2020). Non-targeted metabolomics analysis reveals dynamic changes of volatile and non-volatile metabolites during oolong tea manufacture. Food Res. Int..

[9] Han Z.X., Rana M.M., Liu G.F., Gao M.J., Li D.X., Wu F.G., Li X.B., Wan X.C., Wei S. (2016). Green tea flavour determinants and their changes over manufacturing processes. Food Chem..

[10] Wang Y., Zheng P.C., Liu P.P., Song X.W., Guo F., Li Y.Y., Ni D.J., Jiang C.J. (2018). Novel insight into the role of withering process in characteristic flavor formation of teas using transcriptome analysis and metabolite profiling. Food Chem..

[11] Yang C., Hu Z.Y., Lu M.L., Li P.L., Tan J.F., Chen M., Lv H.P., Zhu Y., Zhang Y., Guo L. (2018). Application of metabolomics profiling in the analysis of metabolites and taste quality in different subtypes of white tea. Food Res. Int..

[12] Chen Y.L., Duan J., Jiang Y.M., Shi J., Peng L.T., Xue S., Kakuda Y. (2011). Production, quality, and biological effects of oolong tea (*Camellia sinensis*). Food Rev. Int..

[13] Yang P., Song H.L., Lin Y.P., Guo T.Y., Wang L.J., Granvogl M., Xu Y.Q. (2021). Differences of characteristic aroma compounds in Rougui tea leaves with different roasting temperatures analyzed by switchable GC-O-MS and GC × GC-O-MS and sensory evaluation. Food Funct..

[14] Liu Z.B., Chen F.C., Sun J.Y., Ni L. (2022). Dynamic changes of volatile and phenolic components during the whole manufacturing process of Wuyi rock tea (Rougui). Food Chem..

[15] Guo X.Y., Song C.K., Ho C.T., Wan X.C. (2018). Contribution of L-theanine to the formation of 2,5-dimethylpyrazine, a key roasted peanutty flavor in oolong tea during manufacturing processes. Food Chem..

[16] Guo X.Y., Ho C.T., Wan X.C., Zhu H., Liu Q., Wen Z. (2021). Changes of volatile compounds and odor profiles in Wuyi rock tea during processing. Food Chem..

[17] Zhang N., Jing T.T., Zhao M.Y., Jin J.Y., Xu M.J., Chen Y.X., Zhang S.R., Wan X.C., Schwab W., Song C.K. (2019). Untargeted metabolomics coupled with chemometrics analysis reveals potential non-volatile markers during oolong tea shaking. Food Res. Int..

[18] Hollman P.C.H., Arts I.C.W. (2000). Flavonols, flavones and flavanols-nature, occurrence and dietary burden. J. Sci. Food Agr..

[19] Liu X.W., Le Bourvellec C., Guyot S., Renard C.M.G.C. (2021). Reactivity of flavanols: Their fate in physical food processing and recent advances in their analysis by depolymerization. Compr. Rev. Food Sci. Food Saf..

[20] Hu S., He C., Li Y.C., Yu Z., Chen Y.Q., Wang Y.M., Ni D.J. (2021). Changes of fungal community and non-volatile metabolites during pile-fermentation of dark green tea. Food Res. Int..

[21] Fan F.Y., Huang C.S., Tong Y.L., Guo H.W., Zhou S.J., Ye J.H., Gong S.Y. (2021). Widely targeted metabolomics analysis of white peony teas with different storage time and association with sensory attributes. Food Chem..

[22] Koshiishi C., Crozier A., Ashihara H. (2001). Profiles of purine and pyrimidine nucleotides in fresh and manufactured tea leaves. J. Agric. Food Chem..

[23] Zeng L.T., Zhou Y., Gui J.D., Fu X.M., Mei X., Zhen Y.P., Ye T.X., Du B., Dong F., Watanabe N. (2016). Formation of volatile tea constituent indole during the oolong tea manufacturing process. J. Agric. Food Chem..

[24] Huang H., Yao Q.Y., Xia E.H., Gao L.Z. (2018). Metabolomics and transcriptomics analyses reveal nitrogen influences on the accumulation of flavonoids and amino acids in young shoots of tea plant (*Camellia sinensis* L.) associated with tea flavor. J. Agric. Food Chem..

[25] Tang D.D., Liu M.Y., Zhang Q.F., Ma L.F., Shi Y.Z., Ruan J.Y. (2020). Preferential assimilation of NH_4_^+^ over NO_3_^−^ in tea plant associated with genes involved in nitrogen transportation, utilization and catechins biosynthesis. Plant Sci..

[26] Whitfield F.B. (1992). Volatiles from interactions of Maillard reactions and lipids. Crit. Rev. Food Sci. Nutr..

[27] Xiang X.L., Su C., Shi Q.X., Wu J.N., Zeng Z.X., Zhang L.J., Jin S., Huang R.Z., Gao T.X., Song C.W. (2021). Potential hypoglycemic metabolites in dark tea fermented by Eurotium cristatum based on UPLC-QTOF-MS/MS combining global metabolomic and spectrum-effect relationship analyses. Food Funct..

[28] Mengesha A.E., Bummer P.M. (2010). Simple chromatographic method for simultaneous analyses of phosphatidylcholine, lysophosphatidylcholine, and free fatty acids. AAPS PharmSciTech.

[29] Zheng Z.P., Ma J.Y., Cheng K.W., Chao J.F., Zhu Q., Chang R.C.C., Zhao M., Lin Z.X., Wang M.F. (2010). Sulfur-containing constituents and one 1H-pyrrole-2-carboxylic acid derivative from pineapple [*Ananas comosus* (L.) Merr.] fruit. Phytochemistry.

[30] Li H.H., Luo L.Y., Ma M.J., Zeng L. (2018). Characterization of volatile compounds and sensory analysis of jasmine scented black tea produced by different scenting processes. J. Food Sci..

[31] Lichman B.R. (2021). The scaffold-forming steps of plant alkaloid biosynthesis. Nat. Prod. Rep..

[32] Fahmy H.H., Soliman G.A. (2001). Synthesis of new salicylamide derivatives with evaluation of their antiinflammatory, analgesic and antipyretic activities. Arch. Pharm. Res..

[33] Mohapatra S.R., Sadik A., Sharma S., Poschet G., Gegner H.M., Lanz T.V., Lucarelli P., Klingmüller U., Platten M., Heiland I. (2021). Hypoxia routes tryptophan homeostasis towards increased tryptamine production. Front. Immunol..

[34] Back K., Tan D.X., Reiter R.J. (2016). Melatonin biosynthesis in plants: Multiple pathways catalyze tryptophan to melatonin in the cytoplasm or chloroplasts. J. Pineal Res..

[35] Balakrishna P., George S., Hatoum H., Mukherjee S. (2021). Serotonin pathway in cancer. Int. J. Mol. Sci..

[36] Silván J.M., van de Lagemaat J., Olano A., Del Castillo M.D. (2006). Analysis and biological properties of amino acid derivates formed by Maillard reaction in foods. J. Pharmaceut. Biomed..

[37] Foley P.B. (2019). Dopamine in psychiatry: A historical perspective. J. Neural Transm..

[38] Ma C.H., Hung Y.C. (2020). Effect of brewing conditions using a single-serve coffee maker on black tea (*Lapsang Souchong*) quality. Food Sci. Nutr..

[39] Hayat J., Akodad M., Moumen A., Baghour M., Skalli A., Ezrari S., Belmalha S. (2020). Phytochemical screening, polyphenols, flavonoids and tannin content, antioxidant activities and FTIR characterization of *Marrubium vulgare* L. from 2 different localities of Northeast of Morocco. Heliyon.

[40] Chen W., Gong L., Guo Z.L., Wang W.S., Zhang H.Y., Liu X.Q., Yu S.B., Xiong L.Z., Luo J. (2013). A novel integrated method for large-scale detection, identification, and quantification of widely targeted metabolites: Application in the study of rice metabolomics. Mol. Plant.

[41] Chen C.J., Chen H., Zhang Y., Thomas H.R., Frank M.H., He Y.H., Xia R. (2020). TBtools: An integrative toolkit developed for interactive analyses of big biological data. Mol. Plant.

