# Supplementary material for: Directed Accumulation of Nitrogen Metabolites through Processing Endows Wuyi Rock Tea with Singular Qualities
Source: Molecules. 2022 May 19;27(10):3264. doi: 10.3390/molecules27103264 (PMC9147623; doi:10.3390/molecules27103264)
Supplement: Supplementary file 1 [file molecules-27-03264-s001.zip › molecules-1731409-ESI.pdf]

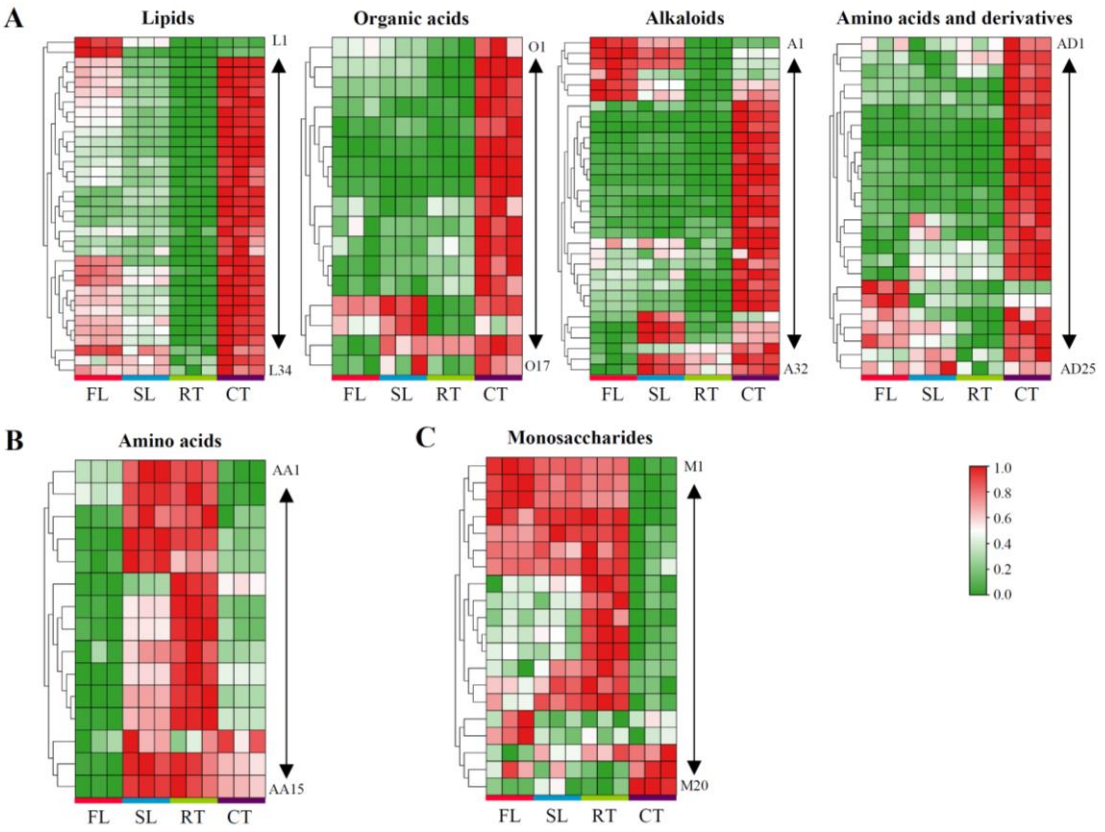

Supplementary Figure S1. Heat maps of specific metabolites. (**A**) N metabolites exhibiting significant increases in concentration during the roasting stage. L1-L34, O1-O17, A1-A32 and AD1-AD25 denote the number of lipids, organic acids, alkaloids, and amino acids and amino acid derivatives, respectively. (**B**) AA1-AA15 denote the number of amino acids significantly increased in the rotation stage. (**C**) M1-M20 denote the number of monosaccharides significantly altered through the three processing stages of Wuyi rock tea production. Information on the metabolites graphed above is detailed in Supplemental Table 1. FL: fresh leaves; SL: spread leaves; RT: raw tea; CT: commercial tea.

Supplementary Table S1. Detailed information on metabolites exhibited in Supplementary Figure S1A.

Number ID	Compounds	Class	Molecular Weight (Da)	Formula	CAS	KEGG ID
L1	LysoPC 18:1(2n isomer)	Lipids	5.21E+02	C26H52NO7P	-	--
L2	LysoPC 19:0	Lipids	5.37E+02	C27H56NO7P	108273-88-7	--
L3	LysoPC 18:2(2n isomer)	Lipids	5.19E+02	C26H50NO7P	-	--
L4	LysoPC 18:2	Lipids	5.19E+02	C26H50NO7P	-	--
L5	LysoPC 17:2	Lipids	5.05E+02	C25H48NO7P	-	--
L6	LysoPC 20:2	Lipids	5.47E+02	C28H54NO7P	-	--
L7	LysoPC 20:3	Lipids	5.45E+02	C28H52NO7P	1199257-41-4	--
L8	LysoPC 16:1	Lipids	4.93E+02	C24H48NO7P	76790-27-7	--
L9	LysoPC 16:1(2n isomer)	Lipids	4.93E+02	C24H48NO7P	-	--
L10	LysoPC 16:0(2n isomer)	Lipids	4.95E+02	C24H50NO7P	-	--
L11	LysoPC 18:3	Lipids	5.17E+02	C26H48NO7P	-	--
L12	LysoPC 18:3(2n isomer)	Lipids	5.17E+02	C26H48NO7P	-	--
L13	LysoPC 19:1	Lipids	5.35E+02	C27H54NO7P	-	--
L14	LysoPC 17:0	Lipids	5.09E+02	C25H52NO7P	50930-23-9	--
L15	LysoPC 17:0(2n isomer)	Lipids	5.09E+02	C25H52NO7P	-	--
L16	LysoPC 16:2	Lipids	4.91E+02	C24H46NO7P	-	--
L17	LysoPC 16:2(2n isomer)	Lipids	4.91E+02	C24H46NO7P	-	--
L18	LysoPC 19:2	Lipids	5.33E+02	C27H52NO7P	-	--
L19	LysoPC 19:2(2n isomer)	Lipids	5.33E+02	C27H52NO7P	-	--
L20	LysoPC 20:0	Lipids	5.51E+02	C28H58NO7P	108341-80-6	--
L21	LysoPC 18:0(2n isomer)	Lipids	5.23E+02	C26H54NO7P	-	--
L22	LysoPC 18:0	Lipids	5.23E+02	C26H54NO7P	19420-57-6	--
L23	LysoPC 18:1	Lipids	5.21E+02	C26H52NO7P	-	--
L24	LysoPC 15:1	Lipids	4.79E+02	C23H46NO7P	-	--
L25	LysoPC 15:0	Lipids	4.81E+02	C23H48NO7P	108273-89-8	--
L26	LysoPC 15:0(2n isomer)	Lipids	4.81E+02	C23H48NO7P	-	--
L27	LysoPC 17:1	Lipids	5.07E+02	C25H50NO7P	-	--
L28	LysoPC 20:1	Lipids	5.49E+02	C28H56NO7P	-	--
L29	LysoPC 16:0	Lipids	4.95E+02	C24H50NO7P	17364-16-8	--
L30	LysoPC 14:0	Lipids	4.67E+02	C22H46NO7P	20559-16-4	--
L31	LysoPC 20:2(2n isomer)	Lipids	5.47E+02	C28H54NO7P	-	--
L32	LysoPC 19:3	Lipids	5.31E+02	C27H50NO7P	-	--
L33	LysoPC 12:0	Lipids	4.39E+02	C20H42NO7P	20559-18-6	--
L34	LysoPC 20:0(2n isomer)	Lipids	5.51E+02	C28H58NO7P	-	--
O1	5-Aminovaleric acid	Organic acids	1.17E+02	C5H11NO2	660-88-8	C00431
O2	Pyrrole-2-carboxylic acid	Organic acids	1.11E+02	C5H5NO2	634-97-9	C05942
O3	2-Picolinic acid	Organic acids	1.23E+02	C6H5NO2	98-98-6	C10164
O4	L-Pipecolic acid	Organic acids	1.29E+02	C6H11NO2	3105-95-1	C00408
O5	5-Acetamidopentanoic acid	Organic acids	1.59E+02	C7H13NO3	1072-10-2	C03087
O6	Benzamide	Organic acids	1.21E+02	C7H7NO	55-21-0	C09815
O7	2-Amino-3-methoxybenzoic acid	Organic acids	1.67E+02	C8H9NO3	3177-80-8	C05831
O8	4-Guanidinobutyric acid	Organic acids	1.45E+02	C5H11N3O2	463-00-3	C01035
O9	4-Acetamidobutyric acid	Organic acids	1.45E+02	C6H11NO3	3025-96-5	C02946
O10	1-Methylpiperidine-2-carboxylic acid	Organic acids	1.43E+02	C7H13NO2	7730-87-2	--
O11	Imidazole-4-acetic acid	Organic acids	1.26E+02	C5H6N2O2	645-65-8	C02835
O12	Anthranilic acid	Organic acids	1.37E+02	C7H7NO2	118-92-3	C00108
O13	Imidazol-1-yl-acetic acid	Organic acids	1.26E+02	C5H6N2O2	22884-10-2	--
O14	2-(Formylamino)benzoic acid	Organic acids	1.65E+02	C8H7NO3	3342-77-6	C05653
O15	2-Aminoisobutyric acid	Organic acids	1.03E+02	C4H9NO2	62-57-7	C03665
O16	Methyl anthranilate	Organic acids	1.51E+02	C8H9NO2	134-20-3	C20634
O17	Creatine	Organic acids	1.31E+02	C4H9N3O2	57-00-1	C00300
A1	1-beta-D-Arabinofuranosyluracil	Alkaloids	2.44E+02	C9H12N2O6	3083-77-0	C16908
A2	Hypoxanthine	Alkaloids	1.36E+02	C5H4N4O	68-94-0	C00262
A3	Adenine	Alkaloids	1.35E+02	C5H5N5	73-24-5	C00147
A4	5-Methylcytosine	Alkaloids	1.25E+02	C5H7N3O	554-01-8	C02376
A5	Histamine	Alkaloids	1.11E+02	C5H9N3	51-45-6	C00388
A6	Cytosine	Alkaloids	1.11E+02	C4H5N3O	71-30-7	C00380
A7	Indole 3-acetic acid (IAA)	Alkaloids	1.75E+02	C10H9NO2	87-51-4	C00954
A8	Thymine	Alkaloids	1.26E+02	C5H6N2O2	65-71-4	C00178
A9	N-Acetyl-5-hydroxytryptamine	Alkaloids	2.18E+02	C12H14N2O2	1210-83-9	C00978

A10	N-Oleoylethanolamine	Alkaloids	3.25E+02	C20H39NO2	111-58-0	C20792
A11	Tryptamine	Alkaloids	1.60E+02	C10H12N2	61-54-1	C00398
A12	Uracil	Alkaloids	1.12E+02	C4H4N2O2	66-22-8	C00106
A13	6-O-methylguanine	Alkaloids	1.65E+02	C6H7N5O	20535-83-5	--
A14	Methyl L-pyroglutamate	Alkaloids	1.43E+02	C6H9NO3	4931-66-2	--
A15	Guanine	Alkaloids	1.51E+02	C5H5N5O	73-40-5	C00242
A16	1-Methyladenine	Alkaloids	1.49E+02	C6H7N5	5142-22-3	C02216
A17	2-Ethyl-2,6,6-trimethylpiperidin-4-one	Alkaloids	1.69E+02	C10H19NO	133568-79-3	--
A18	1,7-Dimethylxanthine	Alkaloids	1.80E+02	C7H8N4O2	611-59-6	C13747
A19	7-Methylguanine	Alkaloids	1.65E+02	C6H7N5O	578-76-7	C02242
A20	p-Coumaroylagmatine	Alkaloids	2.76E+02	C14H20N4O2	7295-86-5	C04498
A21	1,4-Dihydro-1-Methyl-4-oxo-3-pyridinecarboxamide	Alkaloids	1.52E+02	C7H8N2O2	769-49-3	C05843
A22	1-Methylguanidine	Alkaloids	7.31E+01	C2H7N3	471-29-4	C02294
A23	Salicylamide	Alkaloids	1.37E+02	C7H7NO2	65-45-2	--
A24	Isoguanine	Alkaloids	1.51E+02	C5H5N5O	3373-53-3	--
A25	N-Acetylputrescine	Alkaloids	1.30E+02	C6H14N2O	18233-70-0	C02714
A26	Agmatine	Alkaloids	1.30E+02	C5H14N4	306-60-5	C00179
A27	N-Acetylisatin	Alkaloids	1.89E+02	C10H7NO3	574-17-4	C02172
A28	Indole-3-carboxaldehyde	Alkaloids	1.45E+02	C9H7NO	487-89-8	C08493
A29	4-Hydroxyquinoline	Alkaloids	1.45E+02	C9H7NO	611-36-9	C06343
A30	Theophylline	Alkaloids	1.80E+02	C7H8N4O2	58-55-9	C07130
A31	3-Methylxanthine	Alkaloids	1.66E+02	C6H6N4O2	1076-22-8	C16357
A32	7-Methylxanthine	Alkaloids	1.66E+02	C6H6N4O2	552-62-5	C16353
AD1	N-acetyl-beta-alanine	Amino acids and derivatives	1.31E+02	C5H9NO3	3025-95-4	C01073
AD2	1-Methylhistidine	Amino acids and derivatives	1.69E+02	C7H11N3O2	332-80-9	--
AD3	4-Hydoxy-L-Isoleucine	Amino acids and derivatives	1.47E+02	C6H13NO3	781658-23-9	--
AD4	Cycloleucine	Amino acids and derivatives	1.29E+02	C6H11NO2	52-52-8	C03969
AD5	L-Cyclopentylglycine	Amino acids and derivatives	1.43E+02	C7H13NO2	2521-84-8	--
AD6	Trimethyllysine	Amino acids and derivatives	1.88E+02	C9H20N2O2	23284-33-5	C03793
AD7	L-Tyramine	Amino acids and derivatives	1.37E+02	C8H11NO	51-67-2	C00483
AD8	L-Tyrosine methyl ester	Amino acids and derivatives	1.95E+02	C10H13NO3	1080-06-4	C03404
AD9	5-Oxo-L-Proline	Amino acids and derivatives	1.29E+02	C5H7NO3	98-79-3	C01879
AD10	Homoarginine	Amino acids and derivatives	1.88E+02	C7H16N4O2	156-86-5	C01924
AD11	N-(3-Indolylacetyl)-L-alanine	Amino acids and derivatives	2.46E+02	C13H14N2O3	57105-39-2	--
AD12	N-Monomethyl-L-arginine	Amino acids and derivatives	1.88E+02	C7H16N4O2	17035-90-4	C03884
AD13	5-Oxoproline	Amino acids and derivatives	1.29E+02	C5H7NO3	149-87-1	C02237
AD14	N-Methyl-Trans-4-Hydroxy-L-Proline	Amino acids and derivatives	1.45E+02	C6H11NO3	4252-82-8	--
AD15	N-Acetyl-L-tyrosine	Amino acids and derivatives	2.23E+02	C11H13NO4	537-55-3	--
AD16	(2S,3R,4S)-4 -Hydroxyisoleucine	Amino acids and derivatives	1.47E+02	C6H13NO3	55399-93-4	--
AD17	O-Acetylserine	Amino acids and derivatives	1.47E+02	C5H9NO4	5147-00-2	C00979
AD18	N-Acetyl-L-leucine	Amino acids and derivatives	1.73E+02	C8H15NO3	1188-21-2	C02710
AD19	L-Valyl-L-Leucine	Amino acids and derivatives	2.30E+02	C11H22N2O3	3989-97-7	--
AD20	N-Acetyl-L-glutamic acid	Amino acids and derivatives	1.89E+02	C7H11NO5	1188-37-0	C00624
AD21	Cyclo(Pro-Phe)	Amino acids and derivatives	2.44E+02	C14H16N2O2	3705-26-8	C11847
AD22	Cyclo(D-Leu-L-Pro)	Amino acids and derivatives	2.10E+02	C11H18N2O2	36238-67-2	--

AD23	Cyclo(D-Phe-L-Pro)	Amino acids and derivatives	2.44E+02	C14H16N2O2	26488-24-4	--
AD24	L-Ornithine	Amino acids and derivatives	1.32E+02	C5H12N2O2	70-26-8	C00077
AD25	N-Acetyl-L-Glutamine	Amino acids and derivatives	1.88E+02	C7H12N2O4	2490-97-3	--
AA1	L-Leucine	Amino acids	1.31E+02	C6H13NO2	61-90-5	C00123
AA2	L-Isoleucine	Amino acids	1.31E+02	C6H13NO2	73-32-5	C00407
AA3	L-Asparagine	Amino acids	1.32E+02	C4H8N2O3	70-47-3	C00152
AA4	L-Valine	Amino acids	1.17E+02	C5H11NO2	72-18-4	C00183
AA5	L-Proline	Amino acids	1.15E+02	C5H9NO2	147-85-3	C00148
AA6	L-Aspartic Acid	Amino acids	1.33E+02	C4H7NO4	56-84-8	C00049
AA7	L-Lysine	Amino acids	1.46E+02	C6H14N2O2	56-87-1	C00047
AA8	L-Glutamine	Amino acids	1.46E+02	C5H10N2O3	56-85-9	C00064
AA9	L-Serine	Amino acids	1.05E+02	C3H7NO3	56-45-1	C00065
AA10	L-Phenylalanine	Amino acids	1.65E+02	C9H11NO2	63-91-2	C00079
AA11	L-Threonine	Amino acids	1.19E+02	C4H9NO3	72-19-5	C00188
AA12	L-Tryptophan	Amino acids	2.04E+02	C11H12N2O2	73-22-3	C00078
AA13	L-Glycine	Amino acids	1.17E+02	C4H7NO3	543-24-8	--
AA14	L-Cysteine	Amino acids	1.35E+02	C4H9NO2S	1187-84-4	C22040
AA15	L-Tyrosine	Amino acids	1.81E+02	C9H11NO3	60-18-4	C00082
M1	D-Fructose	Monosaccharides	1.80E+02	C6H12O6	57-48-7	C05003
M2	D-Glucose	Monosaccharides	1.80E+02	C6H12O6	50-99-7	C00031
M3	D-Mannose	Monosaccharides	1.80E+02	C6H12O6	3458-28-4	C00159
M4	Ribulose-5-Phosphate	Monosaccharides	2.30E+02	C5H11O8P	4300-28-1	C00117
M5	Sedoheptulose	Monosaccharides	2.10E+02	C7H14O7	3019-74-7	C02076
M6	D-Arabinose	Monosaccharides	1.50E+02	C5H10O5	28697-53-2	C00216
M7	DL-Xylose	Monosaccharides	1.50E+02	C5H10O5	25990-60-7	--
M8	Rhamnose	Monosaccharides	1.64E+02	C6H12O5	3615-41-6	C00507
M9	L-Fucose	Monosaccharides	1.64E+02	C6H12O5	2438-80-4	C01019
M10	Glucose-1-Phosphate	Monosaccharides	2.60E+02	C6H13O9P	59-56-3	C00103
M11	D-Fructose 6-Phosphate	Monosaccharides	2.60E+02	C6H13O9P	643-13-0	C00085
M12	D-Glucose 6-Phosphate	Monosaccharides	2.60E+02	C6H13O9P	56-73-5	C00092
M13	D-Threonic Acid	Monosaccharides	1.36E+02	C4H8O5	3909-12-4	C21649
M14	D-Galacturonic Acid	Monosaccharides	1.94E+02	C6H10O7	685-73-4	C00333
M15	D-Ribose	Monosaccharides	1.50E+02	C5H10O5	50-69-1	C00121
M16	2-Dehydro-3-Deoxy-L-Arabinonate	Monosaccharides	1.48E+02	C5H8O5	-	C00684
M17	D-Glucosamine	Monosaccharides	1.79E+02	C6H13NO5	3416-24-8	C00329
M18	D-Fructose-1,6-Biphosphate	Monosaccharides	3.40E+02	C6H14O12P2	488-69-7	C00354
M19	D-Galactose	Monosaccharides	1.80E+02	C6H12O6	59-23-4	C00124
M20	D-Sedoheptuiose-7-Phosphate	Monosaccharides	2.90E+02	C7H15O10P	2646-35-7	--
